# Machine Learning for Predicting Risk of Drug-Induced Autoimmune Diseases by Structural Alerts and Daily Dose

**DOI:** 10.3390/ijerph18137139

**Published:** 2021-07-03

**Authors:** Yue Wu, Jieqiang Zhu, Peter Fu, Weida Tong, Huixiao Hong, Minjun Chen

**Affiliations:** 1 National Center for Toxicological Research, Division of Bioinformatics and Biostatistics, U.S. Food and Drug Administration, Jefferson, AR 72079, USA; Yue.Wu@fda.hhs.gov (Y.W.); Jieqiang.Zhu@fda.hhs.gov (J.Z.); Weida.Tong@fda.hhs.gov (W.T.); Huixiao.Hong@fda.hhs.gov (H.H.); 2 National Center for Toxicological Research, Division of Biochemical Toxicology, U.S. Food and Drug Administration, Jefferson, AR 72079, USA; Peter.Fu@fda.hhs.gov

**Keywords:** drug-induced autoimmune diseases, structural alerts, machine learning, quantum chemistry

## Abstract

An effective approach for assessing a drug’s potential to induce autoimmune diseases (ADs) is needed in drug development. Here, we aim to develop a workflow to examine the association between structural alerts and drugs-induced ADs to improve toxicological prescreening tools. Considering reactive metabolite (RM) formation as a well-documented mechanism for drug-induced ADs, we investigated whether the presence of certain RM-related structural alerts was predictive for the risk of drug-induced AD. We constructed a database containing 171 RM-related structural alerts, generated a dataset of 407 AD- and non-AD-associated drugs, and performed statistical analysis. The nitrogen-containing benzene substituent alerts were found to be significantly associated with the risk of drug-induced ADs (odds ratio = 2.95, *p* = 0.0036). Furthermore, we developed a machine-learning-based predictive model by using daily dose and nitrogen-containing benzene substituent alerts as the top inputs and achieved the predictive performance of area under curve (AUC) of 70%. Additionally, we confirmed the reactivity of the nitrogen-containing benzene substituent aniline and related metabolites using quantum chemistry analysis and explored the underlying mechanisms. These identified structural alerts could be helpful in identifying drug candidates that carry a potential risk of drug-induced ADs to improve their safety profiles.

## 1. Introduction

Autoimmune disease (AD) is a clinical condition that occurs when the immune system mistakenly attacks one’s own normal cells. More than 100 types of autoimmune diseases have been identified, affecting about 7–9% of the population [1], who are mostly female. The annual medical cost of treating ADs in the United States (U.S.) healthcare system was estimated to be greater than USD 100 billion [2].

ADs often are triggered by environmental stimuli in susceptible individuals with genetic predisposition [1,2]. Drugs are known to account for a significant subset of common clinical ADs. For example, 10% of lupus erythematosus cases and 12–17% of autoimmune hepatitis cases were estimated to be caused by drugs [3,4]. Notably, recent reports suggested that ADs are common conditions following COVID-19, which could be attributable to the acute respiratory distress syndrome or medical treatment [5,6]. The latency of drug-induced ADs can be months or even years [7] and sometimes leads to serious or fatal outcomes. Some drugs, such as nitrofurantoin, minocycline, and methyldopa, are frequently identified as causes of drug-induced ADs [8,9]; however, no effective approach exists for assessing the capability of drugs to induce ADs.

Reactive metabolite formation is a well-documented underlying mechanism for drug-induced ADs [10,11]. Cytochrome P450 enzymes are not only sources but also targets of reactive metabolites, and the covalent binding of drug metabolism enzymes may result in neoantigen formation, triggering an autoimmune response [12]. A compound’s potential to form toxic reactive metabolites is largely determined by its chemical structure, and some substructures can serve as structural alerts for high reactivity and drug toxicity [13,14]. Structural alerts provide mechanistic explanations and insights, which in turn can be used to guide necessary structural changes [15]. These alerts were usually considered as information suggesting the mode of action, but rather a confident link to toxicity [15,16]. Due to their easy application and interpretation, structural alerts have been used to identify compounds with certain toxicities, such as carcinogenicity and hepatotoxicity, in drug development [14,15,17].

Toxicity assessment using in silico approaches has been acceptable to regulatory authorities as complementary information to conventional animal studies for decision making [15,17,18,19]. Guidance documents have been published to specify the incorporation of in silico research on genotoxicity of drug impurities by regulatory agencies including the FDA and the European Medicines Agency [18,19]. Combined with quantitative structure activity relationship (QSAR) models and/or chemical biological read-across models, structural alerts were used in the computational toxicology software MC4PC and MDL-QSAR. MC4PC mines structural alerts by comparing active and inactive compounds using a hierarchical statistic and calculating physicochemical descriptors to build local QSAR models. MDL-QSAR, on the other hand, predicts mutagenicity based on two-dimensional molecular descriptors including E-state descriptors [20]. These methods have been adopted and enhanced by the FDA under the Cooperative Research and Development Agreement between the FDA and industry, for the prediction of carcinogenic activities of drug impurities [20]. In Europe, structural alerts have been accepted for use under the Registration, Evaluation, Authorization, and Restriction of Chemicals (REACH) regulation passed by the European Council and European Parliament [15,21]. The further use of state-of-the-art artificial intelligence technologies such as machine learning and deep learning in the area of QSAR modeling has been shown to be promising in predicting drug toxicity [22].

Metabolism of drugs containing certain chemical structures can generate metabolites that are chemically reactive with hundreds of structural alerts reported [13,14,15]. However, not all of these alerts are created equally; although widely accepted in chemical toxicity assessment, structural alerts are considered overly sensitive in predicting adverse drug reactions (ADRs) when used as the only predictor [14,16]. It is common that both toxic and safe drugs contain the same structural alert, which leads to a high false rate of using structural alerts alone [14]. Thus, it is important to discriminate specific structural alerts that could robustly signal the risk of toxicity endpoints. For drug-induced ADs, no structural alerts are reported despite the known correlation between drug-induced immune responses and reactive metabolites. In this study, we aim to develop a workflow to examine the association between structural alerts and drugs-induced ADs and improve toxicological prescreening tools for drug development. A mechanism-driven bioinformatic approach was used to evaluate 171 published structural alerts for reactive metabolite formation on a large drug-induced ADs dataset including 407 drugs to identify structural alerts that could be used to flag the potential risk of drug-induced ADs. By using a daily dose combined with structural alerts as features, we established a predictive model based on machine learning to facilitate the risk assessment of drug-induced ADs. The underlying molecular mechanisms of drug-induced ADs associated with reactive metabolites and the strategies for mitigating potential toxicity risks in drug development were also discussed.

## 2. Materials and Methods

**Figure 1:**
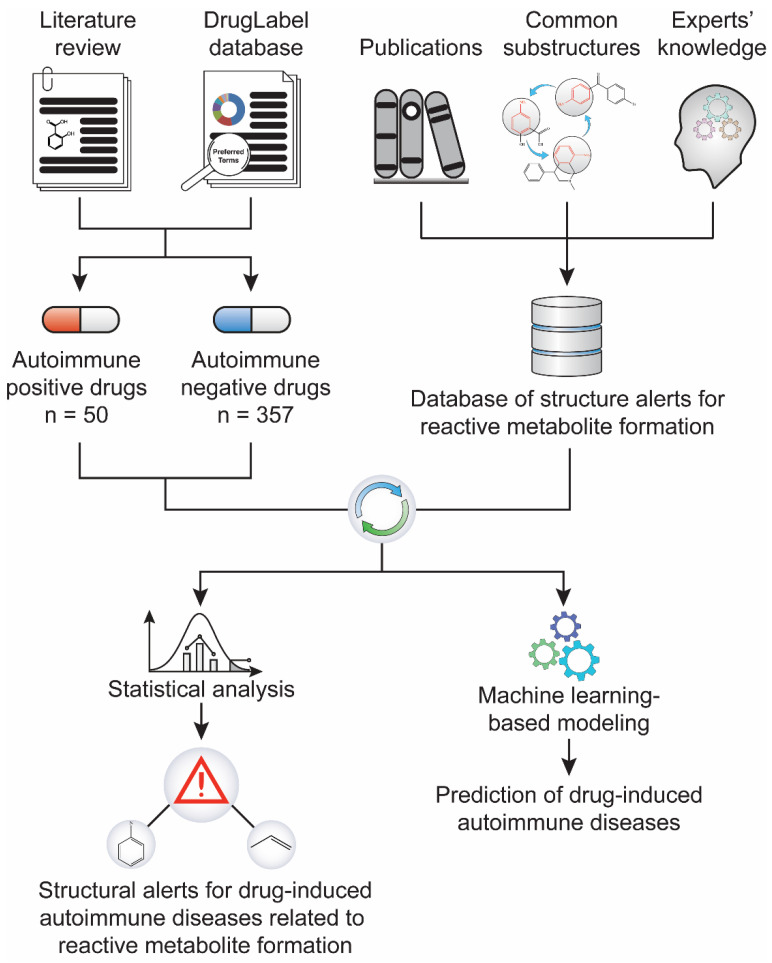
Study workflow. A dataset of 407 drugs, including 50 positives for drug-induced autoimmune disease (AD) and 357 negatives, was compiled through literature text mining and the FDA’s drug label database. In parallel, a library of 171 structural alerts for reactive metabolite formation was collected from literature. The statistical association between the reactive metabolite-related structural alerts and drug-induced ADs was analyzed.

We first built a database by collecting published structural alerts for reactive metabolite formation from the literature and generated a dataset containing both drugs with determined potential to induce ADs (AD-positive) and those not associated with ADs (AD-negative) (Figure 1). We then performed statistical analyses to identify the association between the structural alerts and the risk of drug-induced ADs, and daily dose was co-factored to strengthen the relationship. Further, a machine-learning-based predictive model was established using structural alerts and daily dose as input features. Quantum chemistry analysis was also used to quantify the reactivity of the example substructure aniline and its reactive metabolites.

### 2.1. Collection of Drugs Associated or Not Associated with ADs

To test the association of structural alerts with drug-induced Ads, we first examined the potential of drugs to induce ADs. The text mining software Linguamatics (IQVIA. Marlborough, MA) was used to conduct a full-text search for the 26 AD-related MedDRA terms (Supplemental Table S1) in the Drug Label database (https://dailymed.nlm.nih.gov/dailymed/ (accessed on 20 October 2020)). The most commonly identified ADs in the Drug Label database are blood-affecting disorders including thrombocytopenia, hemolytic anemia, and vasculitis, followed by disorders that affect the skin or other organs (Supplemental Table S1).

If evidence of ADs could not be found in the drug label, the drug was defined as AD-negative. Consequently, 588 drugs were not associated with any of the 26 MedDRA terms. Furthermore, herbal and dietary supplements, biologics, over-the-counter drugs without daily dose information, topical drugs, and medical imaging reagents were excluded, leaving 357 oral or injected drugs as AD-negative.

For drugs with AD-related terms found in the Drug Label database, we examined their evidences associated with drug-induced ADs in literature. Thirty-one drugs were confirmed to have known association with drug-induced ADs, which were summarized in review articles (Supplemental Table S2), and additional nineteen drugs were found with evidence of drug-induced ADs reported by multiple institutes from different countries (Supplemental Table S2). In sum, 50 drugs were determined as AD-positive, supported by both drug labeling and literature. The final dataset contained 407 drugs, which were annotated with a simplified molecular-input line-entry system (SMILES) code.

### 2.2. Development of Structural Alert Database

Since reactive metabolites formation is a common underlying mechanism for drug-induced ADs [10,11], we assumed that structural alerts established for reactive metabolites formation could be used to identify AD-related drugs. These were collected from the literature [13,14,15] and translated into SMILES arbitrary target specification (SMARTS) codes. We then constructed a library of 171 structural alerts for drugs likely to generate reactive metabolites.

### 2.3. Search the Structural Alerts in Chemical Structures

The SMILES-SMART pattern matching function in Rdkit [23], a Python cheminformatics package, was used to search the structural alert library for drugs containing substructures. The 407 drugs were screened against the library. The AD-positive and -negative drugs were considered true positives and true negatives for statistical analysis. High daily dose has been identified as a contributing factor to ADRs [14,16,24], and as was suggested in the literature, we set the cutoff daily dose to ≥100 mg [17].

### 2.4. Development of the Predictive Model Using Machine-Learning Approach

**Figure 2:**
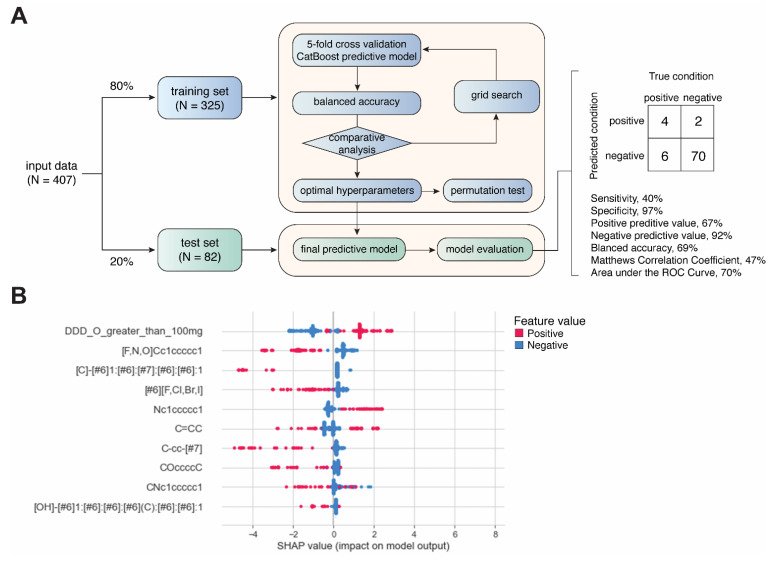
Development of machine-learning-based predictive model. (**A**) Workflow of model development. (**B**) Summary plot showing SHAP values and feature values for top 10 most important features.

Daily dose was included as a categorical feature by using a cutoff of 100 mg/day. Each drug was labeled as either with a daily dose ≥100 mg or <100 mg. The structural alerts were also used as categorical features. The drugs were labeled as presence of non-presences of a matched structural alert across all the candidate structural alerts in our library. The resulting dataset was stratified and split into training (80%, *N* = 325) and test (20%, *N* = 82) sets, which were used to train and evaluate binary classification models generated by CatBoost, respectively [25,26] (Figure 2A). Grid search was performed for hyperparameter tuning. For each set of hyperparameters, 5-fold cross-validation was used for evaluating model performance based on balanced accuracy (i.e., (sensitivity + specificity)/2). Permutation analysis was conducted to determine whether a model performs at chance [27]. Permutated datasets (*N* = 1000) were generated by randomly reshuffling the classification labels (AD-positive or -negative). The performance of the resulting 1000 models was compared with models from 1000 repetitions of 5-fold cross-validations with different sampling. A two-sided *t*-test was used determine the statistical significance of the difference between the results obtained from permutated data and original data. Feature importance and contribution were explored using Shapley additive explanations (SHAP) values [28], which quantifies the contribution of each feature to the prediction made by the model (Figure 2B). All analysis was performed using Python programming. The hyperparameters used for the final CatBoost model are listed in Supplemental Table S3.

### 2.5. Quantum Chemistry Analysis

The General Atomic and Molecular Electronic Structure System (GAMESS) (version 30 June 2019 R1) software (Iowa State University, Ames, IO, United States) [29] was used to calculate quantum chemistry properties, e.g., electron density. Input files were prepared by Avogadro (version 1.2.0) (http://avogadro.cc/ accessed on 27 June 2021) [30]. Geometry optimizations were performed using a global-hybrid meta-NGA functional (MN15) with a 6-31+G (d,p) basis set used for density function theory calculations [31]. The solvent parameter was set to water (pH = 7). Energy levels of the lowest unoccupied molecular orbital (*E*_LUMO_), highest occupied molecular orbital (*E*_HOMO_), and the electron density were used to measure reactive potential for the example substructure aniline and the related reactive metabolites in our analysis [32]. The *E*_LUMO_ and *E*_HOMO_ obtained from quantum chemistry analysis were used to calculate the global hardness η = (*E*_LUMO_ − *E*_HOMO_)/2, chemical potential μ = (*E*_LUMO_ + *E*_HOMO_)/2, and electrophilicity index ω = μ^2^/2η.

## 3. Results

### 3.1. Association between AD-Positive/Negative Drugs and Reactive Metabolites-Related Structural Alerts

To establish the association between structural alerts and a drug’s potential to cause AD, the library of 171 published reactive metabolites-related structural alerts was used to screen for *N* = 50 AD-positive drugs and *N* = 357 AD-negative drugs (Figure 1). We highlighted the top 10 structural alerts, which are most frequently contained in the chemical structures of AD-positive drugs (Table 1). Only one structural alert was found to be statistically associated with AD-positive drugs (odds ratio = 2.95, *p* = 0.0036). This structural alert is a benzene ring with a nitrogen-containing substituent group, which can match to a series of substructures including aniline, anilide, azobenzene, etc.

As a comparison, we combined all structural alerts; if any of these were matched to a drug, the drug was considered to carry a structural alert for reactive metabolites formation. No statistically significant association between combined structural alerts and a risk of drug-induced ADs was observed (*p* = 0.2903), and the false positive rate was 46% (Table 1).

### 3.2. Integration of Structural Alerts with Daily Dose

The use of the nitrogen-containing benzene substituent alert alone still led to a high false positives rate (i.e., 12%); therefore, we further factored daily dose into the analysis. By combining this structural alert and daily dose ≥ 100 mg, the false positive rates dropped significantly, from 12% to 4%, and the positive predictive rate (PPV) increased from 25% to 42% (Table 1 and Table 2). In contrast, the sensitivity showed minimal change, as the number of true positives was much less affected by the daily dose cutoff (≥100 mg) compared to the number of false positives. Of note, although high daily dose alone was associated with drug-induced ADs, the false positive rate was high (39%) (Table 1).

The nitrogen-containing substituent group can be primary, secondary, or tertiary amine. We examined each of these types of substructures and found that primary (odds ratio = 22.72, *p* = 0.0064) and tertiary (odds ratio = 9.81, *p* = 0.0019) amines, not secondary amines (*p* = 0.1692), were significantly associated with AD-positive drugs when co-factoring with daily dose ≥ 100 mg (Table 2). Furthermore, we separated this structural alert into amine and benzene substructures (Table 2). Benzene showed no statistically significant association with AD-related drugs (*p* = 0.8720). Although amines were significantly associated with AD-positive drugs (odds ratio = 3.08, *p* = 0.0004), they exhibited a high false positive rate of 39%. Neither benzene nor amine substructures were of good predictive value, which resulted in much higher false positive rates.

### 3.3. Predictive Modeling Based on Structural Alerts and Daily Dose

Not all the structural alerts contribute equally to the prediction of drug-induced ADs; moreover, other contributing factors such as daily dose could also be important. Therefore, we further developed a machine-learning-based predictive model using structural alerts and daily dose as input features (Figure 2A). After optimization, the final model generated by CatBoost exhibited excellent performance in predicting AD-negative drugs in the test set with specificity of 97% and negative predictive value (NPV) of 92%. In contrast, the prediction of AD-positive drugs was less robust, as the sensitivity was 40%. Overall, the results obtained from the test set showed a balanced accuracy of 69%, Matthews correlation coefficient (MCC) of 47%, and AUC of 70% (Supplemental Figure S1). We also conducted additional permutation test on the training set, and the results showed that the average balanced accuracy from cross-validations was significantly higher than that from permutations (61% vs. 50%, *p* < 0.0001) (Supplemental Figure S2).

Next, we explored the feature importance represented by SHAP values. Intriguingly, only daily dose and nitrogen-containing benzene substituent alert among the top features showed a positive correlation with predicted values, as positive feature values (red) pushed the model towards positive predicted values (higher SHAP values). This is consistent with the association analysis (Figure 2B).

### 3.4. Quantum Chemistry Analysis

**Figure 3:**
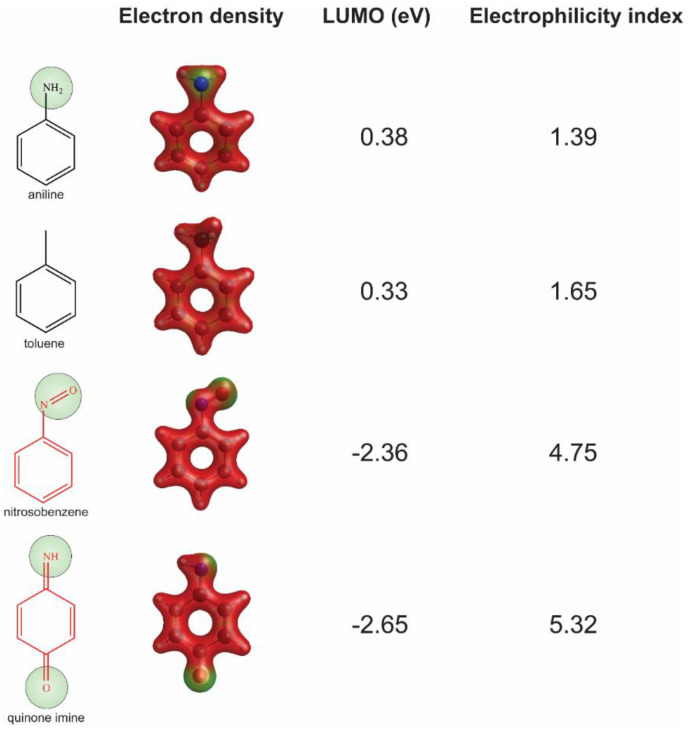
Assessment of reactivity of aniline, nitrosobenzene, quinone imine, and toluene by quantum chemistry analysis. Low electron density areas are shown in green, indicating the potential electrophilicity of the chemicals.

We chose substructure aniline as the abovementioned structural alert, a benzene ring with a nitrogen-containing substituent group. To quantify the reactivity of reactive metabolites, we performed geometry optimizations of aniline and toluene (a paired control compound for aniline) and nitrosobenzene and quinone imine (two reactive metabolites of aniline) [14,33,34]. Their quantum chemistry properties were calculated with the solvent parameter set as water (pH = 7). Low electron density (green) was observed for the electrophilic groups of aniline, nitrosobenzene, and quinone imine (Figure 3). A higher electrophilicity index indicates higher electrophilicity of the compound, while a lower *E*_LUMO_ means lower energy is required for electrons in the nucleophile to occupy the LUMO in the electrophile. The two chemically reactive metabolites, nitrosobenzene and quinone imine, both showed a much higher electrophilicity index and lower *E*_LUMO_ as compared to aniline (Figure 3). However, aniline was found not more reactive than a non-structural alert control toluene, as they showed similar *E*_LUMO_ and electrophilicity index. The significantly enhanced reactivity of reactive metabolites suggested that the aniline toxicity is likely to be mediated by the reactive metabolites but itself. The results from quantum chemistry analysis were corroborated by current knowledge of aniline and its derivatives.

## 4. Discussion

In this study, we constructed a library of structural alerts for reactive metabolites formation and examined the association of the alerts with drug-induced ADs. Substructures of benzene with nitrogen-containing substituent were found to be significantly associated with an increased risk of drug-induced ADs, and by factoring in high daily dose, (≥100 mg) the false positive rate was significantly reduced to 4%, and meanwhile, the positive predictive value was increased from 25% to 42%. Furthermore, we developed a machine-learning-based predictive model by using daily dose and nitrogen-containing benzene substituent alert as the top inputs and achieved the predictive performance of AUC of 70%. Permutation analysis suggests this association is robust and not by chance. We also confirmed the reactivity of the nitrogen-containing benzene substituent aniline and related metabolites using quantum chemistry analysis and explored the underlying mechanisms.

Based on the association analysis and the feature importance evaluation, substructures of benzene with nitrogen-containing substituent were associated with AD-positive drugs and significantly contributed to the model prediction. Aniline derivatives, as examples that match this structural alert, have been reported to induce allergic and autoimmune reactions [35,36]. Drugs with such substructures, including sulfonamides and procainamide, have reportedly caused ADs in humans [37,38,39]. In our dataset, 14 AD-positive drugs contain this structural alert. The chemical structures and their daily doses are shown in Supplemental Figure S3.

Notably, concerns that structural alerts are overly sensitive (i.e., a high number of false positives) in flagging toxic compounds have been reported, and simply avoiding structural alerts for this reason may be too restrictive [14]. Indeed, all the top 10 structural alerts in Table 1 have high false positive rates (6–24%). Some commonly prescribed drugs do contain structural alerts but have low risks of toxicity [14]; for example, half of the top 200 drugs by prescription and sales in 2009 have structural alerts, and many could form reactive metabolites [14]. Therefore, it was proposed that structural alerts should be considered together with other factors when evaluating toxicity risk [14,15,16]. High dose is a significant contributing factor to reactive metabolite-related toxicity [13,14,15]. For instance, 13 of the top 15 small molecule drugs by annual sales in the U.S. market contain at least one structural alert [14], but most of their recommended daily doses are relatively low (<100 mg), which reduces their toxicity risks [14]. Our study confirmed this co-factoring approach, and after taking daily dose into consideration, the false positive rates of the selected structural alerts were dramatically reduced. Of note, the most important feature for the model prediction was daily dose.

Using aniline as an example, two metabolic pathways for bioactivation of benzene with nitrogen-containing substituent leading to toxicity have been proposed for reactive metabolite formation [36,37,39] (Supplemental Figure S4). Aniline can be oxidized, catalyzed by CYPs, at the aromatic amine group, leading to the generation of nitrosobenzene, or undergoing electrophilic substitution in the benzene ring to form quinone imine. Both nitrosobenzene and quinone imine are highly electrophilic and, thus, are prone to forming protein adducts by reacting with nucleophilic residues, especially cysteines, leading to toxicity [34,40].

Procainamide (Supplemental Figure S3), an antiarrhythmic, has been associated with drug-induced autoimmune diseases [7,41]. The incidence was estimated to be as high as 20% [7]. Procainamide contains a benzene with nitrogen-containing substituent substructure, which can be metabolized into reactive derivatives, hydroxylamine and nitrosobenzene [42,43,44,45]. Animal studies showed that injection of procainamide hydroxylamine into the thymus resulted in the disruption of central T cell tolerance and the initiation of systemic autoimmunity [46,47], in which the production of anti-(H2A-H2B)-DNA autoantibodies, a hallmark of patients with drug-induced lupus, was observed. Furthermore, the results from popliteal lymph node assay in mice showed that T cells only respond to the reactive metabolites of aniline generated by white bone marrow cells, but not to the prohapten aniline itself [34]. Moreover, peripheral T cells could acquire autoreactivity through hypomethylation of DNA [7,41,48]. Procainamide has been reported to inhibit DNA methylation, leading to lymphocyte activation, and the induction of autoimmunity is likely due to the overexpression of leukocyte-function-associated antigen-1 (LFA-1) caused by hypomethylation of DNA in T cells [49].

Quantum chemistry properties have been useful in assessing reactive potential for structural alerts or chemical entities [32,50]. The culprit reactive metabolites, nitrobenzene and quinone imine, are highly reactive, as indicated by the quantum chemistry properties. Our results suggest that quantum chemistry analysis could be a useful tool for evaluating the reactivity of chemicals or their metabolites and providing information for the assessment of toxicity.

In drug development, chemicals with structural alerts were considered for lead optimization or mitigation. Experimental tests for toxicity (e.g., glutathione trapping, protein covalent binding experiments) should be prioritized. Certain strategies could be used to mitigate potential toxicity risks, including modifying substructures to resist metabolism and/or lowering dose by improving pharmacokinetic properties [51].

For instance, clozapine was reported to be associated with drug-induced ADs [52,53] and can form an iminium reactive metabolite that could covalently bind to glutathione or other proteins [54] (Supplemental Figure S5). Reactive metabolite formation is dependent on the nitrogen that bridges the two rings [55]. In quetiapine, this nitrogen is replaced by a sulfur atom (blue), which removes the reactive metabolite formation, even though the daily dose of quetiapine (400 mg) is higher than that for clozapine (300 mg). Olanzapine can also form an iminium reactive metabolite but exhibits an improved safety profile in comparison to clozapine. This is likely due to the low required daily dose (10 mg) of olanzapine, which is attributable to the optimized substructure (blue). Both quetiapine and olanzapine are among the top-selling drugs in the U.S.

Another drug, nefazodone, can form a quinone-imine reactive metabolite, which is highly reactive to the cysteine in glutathione or other proteins [56] (Supplemental Figure S6). Aripiprazole also carries a chloroaniline substructure (red) very close to that of nefazodone, which can form reactive metabolites. However, the daily dose of aripiprazole is much lower (15 mg), due to dramatically improved pharmacokinetics [57] compared to nefazodone (400 mg). Aripiprazole has a significantly improved safety profile and is ranked among the top drugs in sales.

We acknowledge the following limitations of the current study. The data used in this study mainly focused on approved drugs in the market and yet did not include failed drug candidates during clinical trials, withdrawn drugs from the market, or well-characterized allergenic natural products. This limited training scope could render the developed method for toxicity prediction not generalizing well to a broader chemical space during drug development. Although expanding training data to failed/withdrawn drugs and natural products is not a trivial task as such, data are often not readily accessible; it would provide more comprehensive information to improve the toxicological prescreening method and reduce potential bias. Furthermore, the structural alerts were collected from seminal reviews due to their commonly known activities in forming reactive metabolites, which is one of the underlying mechanisms of drug-induced ADs. Additional structural alerts can be included in the future to improve the diversity of the structural alert library. For example, methylcatechol, which has similar chemical functionality to the primary irritant urushiol in poison ivy, could flag risk of flavonoids-derived drug candidates and phenylethyl resorcinol, a cosmetic skin-lightener increasingly implicated in contact allergic dermatitis [58,59]. Acyl halides in drug metabolites could also suggest a toxicity risk of the precursor drugs [60,61]. Overall, the results obtained from the current dataset suggests that this approach is promising and could be legitimately powerful with a broader dataset.

Another limitation is that the training data were imbalanced on AD-positive and -negative. Developing machine-learning models using such an imbalanced dataset could be challenging. There are many methods of handling imbalanced classification problems such as data imputation/removal and using balanced accuracy or MCC as the evaluation metric during model optimization. Lovric et al. systematically evaluated the quality metrics in imbalanced scenarios and suggested MCC to be one of most suitable metrics to evaluate model performance [62]. Despite that balanced accuracy was used as the grid search scoring function in the current study, our model showed a MCC of 0.47 when evaluated using test data. In addition to structural alerts, other features such as fingerprints and physicochemical descriptors can be used to develop predictive models. Our analysis from the model developed using two-dimensional descriptors generated by MOLD2 [63] showed comparable performance with a MCC of 0.42. We chose to use structural alerts associated with reactive metabolite formation from a mechanism-based perspective, and also because structural alerts are relatively more intuitive to interpret in terms of guiding structure optimization. CatBoost, a leading machine-learning algorithm, was used for model development because (i) it transforms categorical features into numeric values using various statistics, (ii) a novel gradient-boosting algorithm is used to reduce bias, (iii) the performance is comparable, if not better, to other boosting libraries such as XGBoost and LightGBM, and (iv) GPU computing is supported for fast model training [25,26]. Of note, some other machine-learning methods (e.g., random forest, support vector machine, and neural networks) were not tested in this study and could potentially improve the result.

## 5. Conclusions

As an increasing number of drug candidates are evaluated in clinical trials, the number and spectrum of drug-induced AD cases has been widely expanding [2,41]. The investigated structural alerts and the developed predictive model can be helpful in identifying drug candidates with potential risks of drug-induced ADs and optimizing chemical structures to avoid potential liability of causing ADs in humans.

## Figures and Tables

**Table 1:** Ten structural alerts most frequently contained in the chemical structures of autoimmune disease (AD)-positive drugs.

Structural Alerts	Description	Number of Matched Drugs		Sensitivity	PPV	FPR	OR	*p* Value
		AD-Positive	AD-Negative					
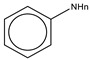	benzene ring with nitrogen-containing substituent	14	42	28%	25%	12%	2.95	*p* = 0.0036
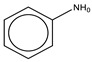	benzene ring with nitrogen-containing substituent (no N-H bond)	7	22	14%	24%	6%	2.51	*p* = 0.0699
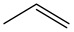	alkenes	12	51	24%	19%	14%	1.92	*p* = 0.0931
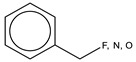	methylbenzene with halogenation at the methyl group	7	87	14%	7%	24%	0.51	*p* = 0.1104
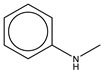	benzene ring with nitrogen-containing substituent (one N-H bond)	8	36	16%	18%	10%	1.72	*p* = 0.2229
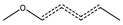	methoxy and methyl group with three aromatic carbon bonds in between	6	30	12%	17%	8%	1.50	*p* = 0.4228
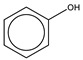	benzene ring with hydroxyl group	6	60	12%	9%	17%	0.68	*p* = 0.5384
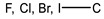	halogenated carbon	11	64	22%	15%	18%	1.31	*p* = 0.5584
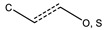	aromatic carbon bond with methyl and O/S groups	6	38	12%	14%	11%	1.16	*p* = 0.8074
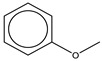	benzene ring with methoxy group-containing substituent	8	60	16%	12%	17%	0.96	*p* > 0.9999
All structural alerts combined		19	166	38%	10%	46%	0.71	*p* = 0.2903
High daily dose (≥100 mg)		36	141	72%	20%	39%	3.94	*p* < 0.0001

**Table 2:** Association between substructures matching benzene with nitrogen-containing substituent and drug-induced ADs after accounting for high daily dose (≥100 mg).

Structural Alerts	Description	Number of Matched Drugs		Sensitivity	PPV	FPR	OR	*p* Value
		AD-Positive	AD-Negative					
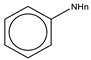 + daily dose ≥ 100 mg	benzene ring with nitrogen-containing substituent	10	14	20%	42%	4%	6.13	*p* = 0.0002
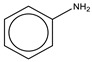 + daily dose ≥ 100 mg	benzene ring with nitrogen-containing substituent (two N-H bond)	3	1	6%	75%	0%	22.72	*p* = 0.0064
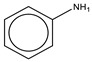 + daily dose ≥ 100 mg	benzene ring with nitrogen-containing substituent (one N-H bond)	3	9	6%	25%	3%	2.47	*p* = 0.1692
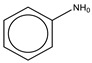 + daily dose ≥ 100 mg	benzene ring with nitrogen-containing substituent (no N-H bond)	5	4	10%	56%	1%	9.81	*p* = 0.0019
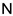 + daily dose ≥ 100 mg	nitrogen-containing compound	33	138	66%	19%	39%	3.08	*p* = 0.0004
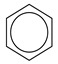 + daily dose ≥ 100 mg	benzene	25	69	50%	27%	19%	4.17	*p* = 0.8720

## Data Availability

Not applicable.

## Supplementary Materials

The following are available online at https://www.mdpi.com/article/10.3390/ijerph18137139/s1. Table S1: Text mining of AD-related MedDRA terms in the Drug Label database; Table S2: Evidence of drug-induced autoimmune disorders for the investigated drugs; Table S3: Hyperparameters tuned for the machine learning-based predictive model; Figure S1: Area under the ROC curve of the developed predictive model; Figure S2: Density distribution of average balanced accuracy scores from permutations and cross-validations; Figure S3: Drugs that contain the structural alert, benzene with nitrogen-containing substituent, and are associated with drug-induced autoimmune diseases; Figure S4. Two bioactivation pathways of aniline, an aromatic amine moiety, in humans. Figure S5: Comparison of clozapine, olanzapine, and quetiapine in terms of reactive metabolite-related toxicity; Figure S6: Comparison of nefazodone and aripiprazole in terms of reactive metabolite-related toxicity.
