# Supplementary material for: Machine Learning for Predicting Risk of Drug-Induced Autoimmune Diseases by Structural Alerts and Daily Dose
Source: Int J Environ Res Public Health. 2021 Jul 3;18(13):7139. doi: 10.3390/ijerph18137139 (PMC8296890; doi:10.3390/ijerph18137139)
Supplement: Supplementary file 1 [file ijerph-18-07139-s001.zip › Supplemental Tables.pdf]

Supplemental Table S1. Text mining of AD-related MedDRA terms in the Drug Label database.

AD-related MedDRA terms	Number of associated drugs
Thrombocytopenia	469
Hemolytic anemia	166
Vasculitis	133
Systemic lupus erythematosus	105
Interstitial lung disease	83
Myopathy	80
Polymyositis	27
Pemphigus	21
Scleroderma	21
Lupus-like syndrome	19
Glomerulonephritis	15
Henoch-Schonlein purpura	15
Thyroiditis	15
Pemphigoid	12
Systemic scleroderma	8
Autoimmune hemolytic anemia	6
Immune-mediated necrotizing myopathy	6
Dermatomyositis	5
Autoimmune hepatitis	3
Pulmonary granuloma	2
Sjogren's syndrome	2
Antiphospholipid syndrome	1
Autoimmune thyroiditis	1
Linear IgA disease	1
Glomerulonephritis rapidly progressive	0
Granulomatous pneumonitis	0

Supplemental Table S2. Evidence of drug-induced autoimmune disorders for the investigated drugs.

Drug names	Reference (PMID)	Evidence level
minocycline	Definite [10720762, 21327704]	Defined by seminal review
hydralazine	Definite [10720762]	Defined by seminal review
procainamide	Definite [10720762]	Defined by seminal review
quinidine	Definite [10720762]	Defined by seminal review
methyldopa	Definite [21327704, 21327704]	Defined by seminal review
nitrofurantoin	Definite [21327704]	Defined by seminal review
acebutolol	Possible [10720762]	Defined by seminal review
allopurinol	Possible [10720762]	Defined by seminal review
captopril	Possible [10720762]	Defined by seminal review

carbamazepine	Possible [10720762]	Defined by seminal review
clonidine	Possible [10720762]	Defined by seminal review
danazol	Possible [10720762]	Defined by seminal review
disopyramide	Possible [10720762]	Defined by seminal review
erythromycin	Possible [10720762]	Defined by seminal review
ethosuximide	Possible [10720762]	Defined by seminal review
griseofulvin	Possible [10720762]	Defined by seminal review
labetalol	Possible [10720762]	Defined by seminal review
lovastatin	Possible [10720762]	Defined by seminal review
metoprolol	Possible [10720762]	Defined by seminal review
pindolol	Possible [10720762]	Defined by seminal review
primidone	Possible [10720762]	Defined by seminal review
promethazine	Possible [10720762]	Defined by seminal review
spironolactone	Possible [10720762]	Defined by seminal review
streptomycin	Possible [10720762]	Defined by seminal review
sulindac	Possible [10720762]	Defined by seminal review
tolmetin	Possible [10720762]	Defined by seminal review
isoniazid	Probable [10720762, 21327704]	Defined by seminal review
chlorpromazine	Probable [10720762]	Defined by seminal review
atorvastatin	Probable [21327704]	Defined by seminal review
diclofenac	Probable [21327704]	Defined by seminal review
propylthiouracil	Probable [21327704]	Defined by seminal review
amiodarone	Possible [3591459; 20525904; 28398380; 10684321]	Reported by multiple institutes and different countries
amoxicillin	Possible [27252926; 25224696]	Reported by multiple institutes and different countries
cefuroxime	Probable [30977941; 23329991; 9015473]	Reported by multiple institutes and different countries
clobazam	Possible [7743738; 26170788; 25690725]	Reported by multiple institutes and different countries
clozapine	Probable [30948402; 28064233; 16670363; 15206677; 27580496; 7870651]	Reported by multiple institutes and different countries
deferoxamine	Possible [1415353; 4050784; 2322425]	Reported by multiple institutes and different countries
disulfiram	Possible [9833921; 24247861]	Reported by multiple institutes and different countries
enalapril	Possible [1973510; 29282395; 26528346]	Reported by multiple institutes and different countries
fluvastatin	Possible [9672281; 16446645; 21503830; 16940892; 17403199]	Reported by multiple institutes and different countries
lisinopril	Possible [30155951; 14754654; 8394798; 11780682]	Reported by multiple institutes and different countries
mesalazine	Possible [1515833; 9385697; 10483021]	Reported by multiple institutes and different countries
pravastatin	Possible [17404733; 16154665; 28831316; 1640970]	Reported by multiple institutes and different countries
quinine	Possible [2297210; 8323089; 9867731; 25414441]	Reported by multiple institutes and different countries
simvastatin	Possible [9743064;15118389; 10628262; 18853127; 22700482]	Reported by multiple institutes and different countries
sulfamethoxazole	Possible [28596226; 29121838; 27357280; 29469749]	Reported by multiple institutes and different countries
sulfasalazine	Possible [27478675; 2894766; 7788172; 27778097]	Reported by multiple institutes and different countries
ticlopidine	Possible [12390069; 20376687; 16961527; 17039105]	Reported by multiple institutes and different countries

trimethoprim	Possible [28596226; 29469749; 31763587]	Reported by multiple institutes and different countries
valproic acid	Possible [2111770; 8112240; 15717181]	Reported by multiple institutes and different countries

Supplemental Table S3. Hyperparameters tuned for the machine learning-based predictive model.

Hyperparameters tuned	Values
**depth**	3
**iterations**	1000
**l2_leaf_reg**	0.001
**leaf_estimation_iterations**	10
**loss_function**	Logloss
**learning_rate**	0.03
