# Supplementary figures and images for: Machine Learning for Predicting Risk of Drug-Induced Autoimmune Diseases by Structural Alerts and Daily Dose
Source: Int J Environ Res Public Health. 2021 Jul 3;18(13):7139. doi: 10.3390/ijerph18137139 (PMC8296890; doi:10.3390/ijerph18137139)

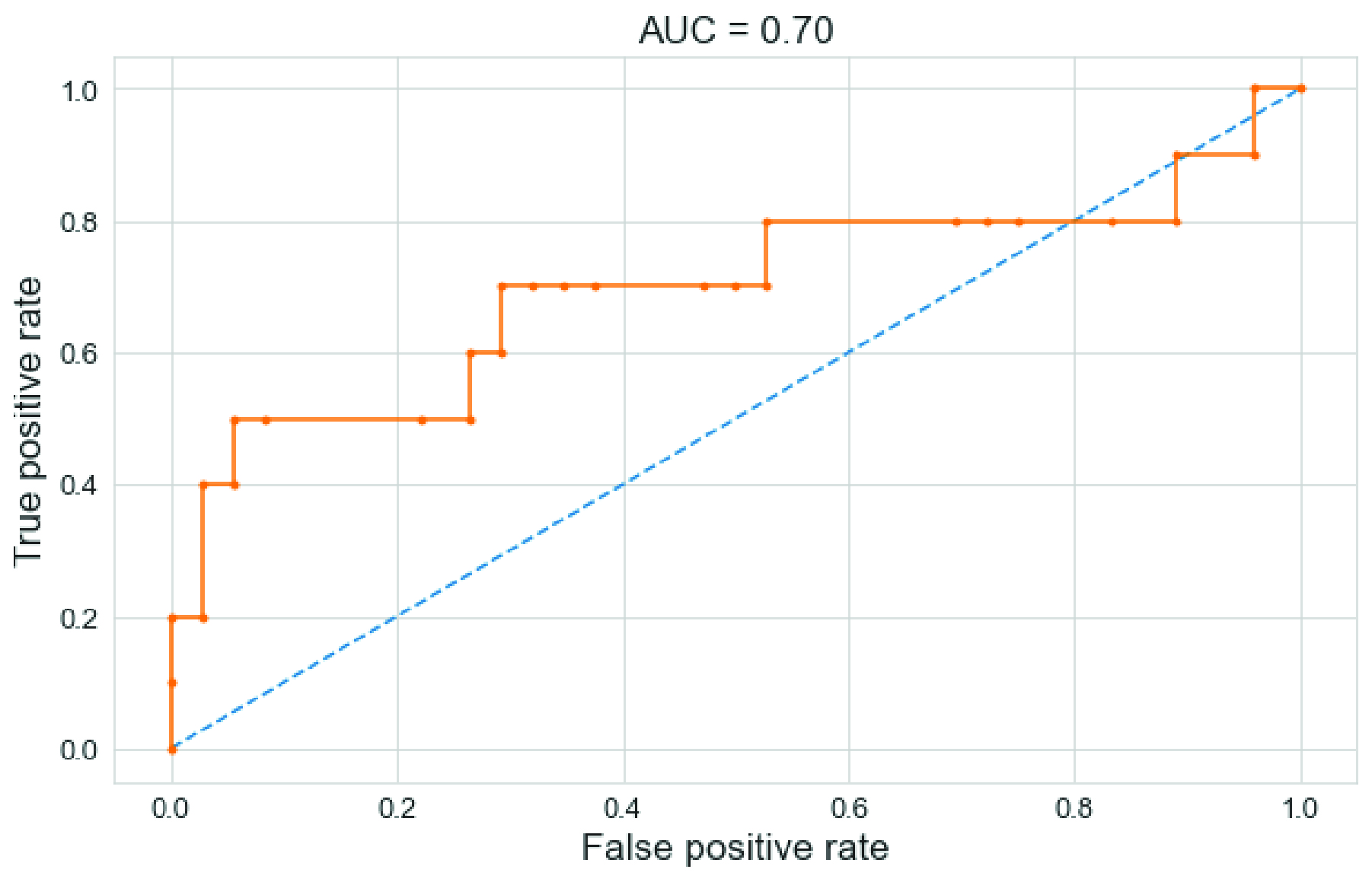

Supplement: Supplementary file 1 [file ijerph-18-07139-s001.zip › Supplemental Figure S1.jpg]

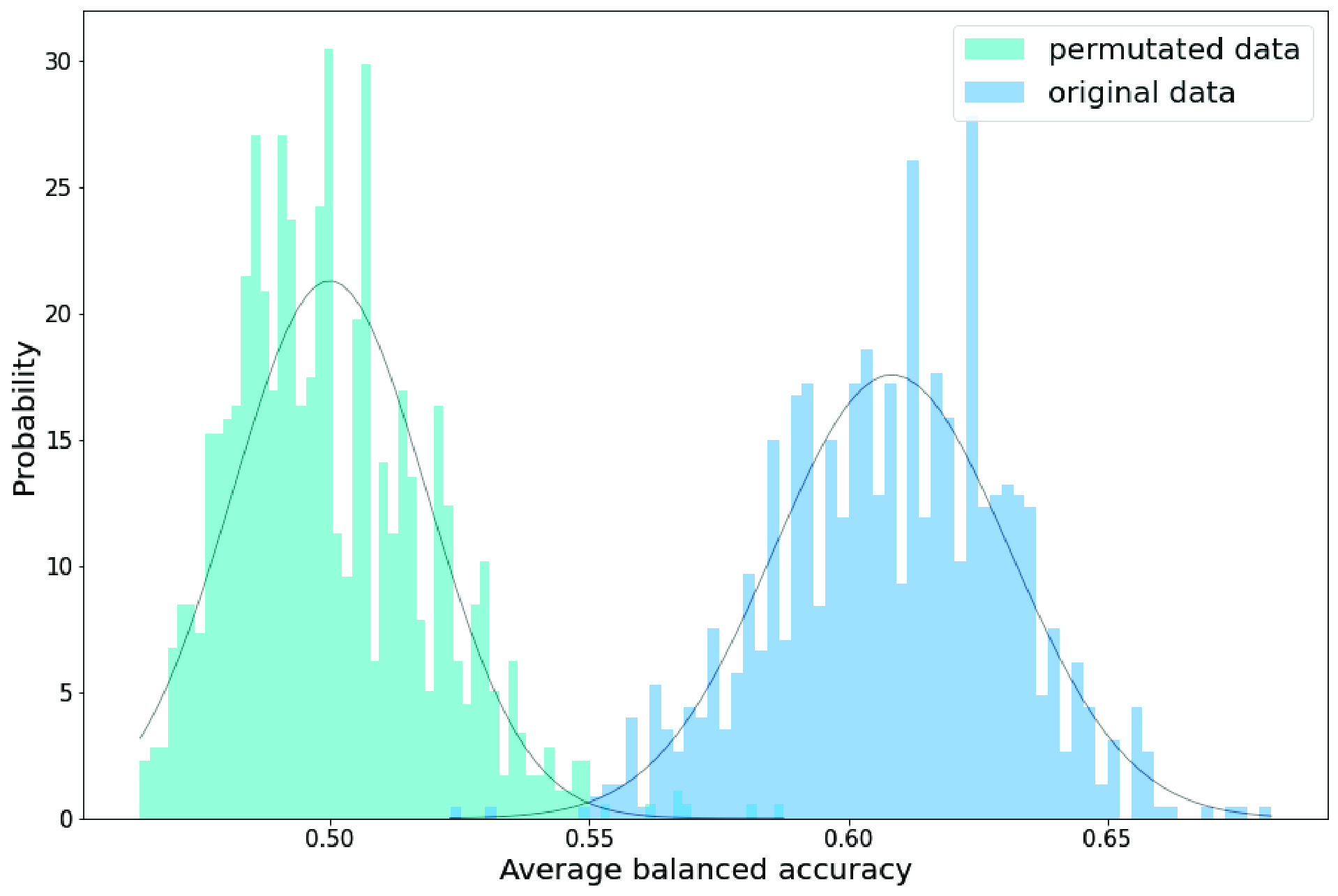

Supplement: Supplementary file 1 [file ijerph-18-07139-s001.zip › Supplemental Figure S2.jpg]

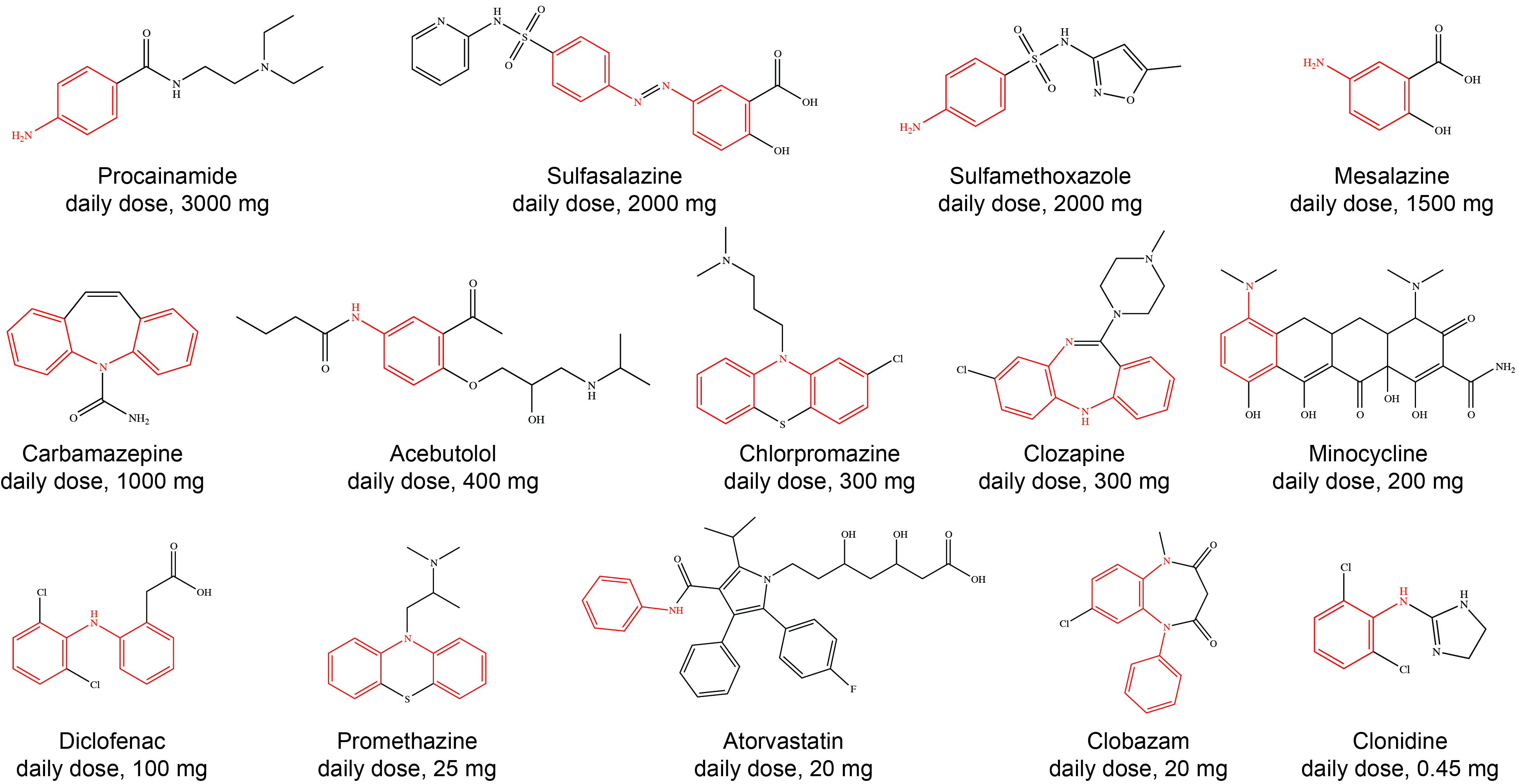

Supplement: Supplementary file 1 [file ijerph-18-07139-s001.zip › Supplemental Figure S3.jpg]

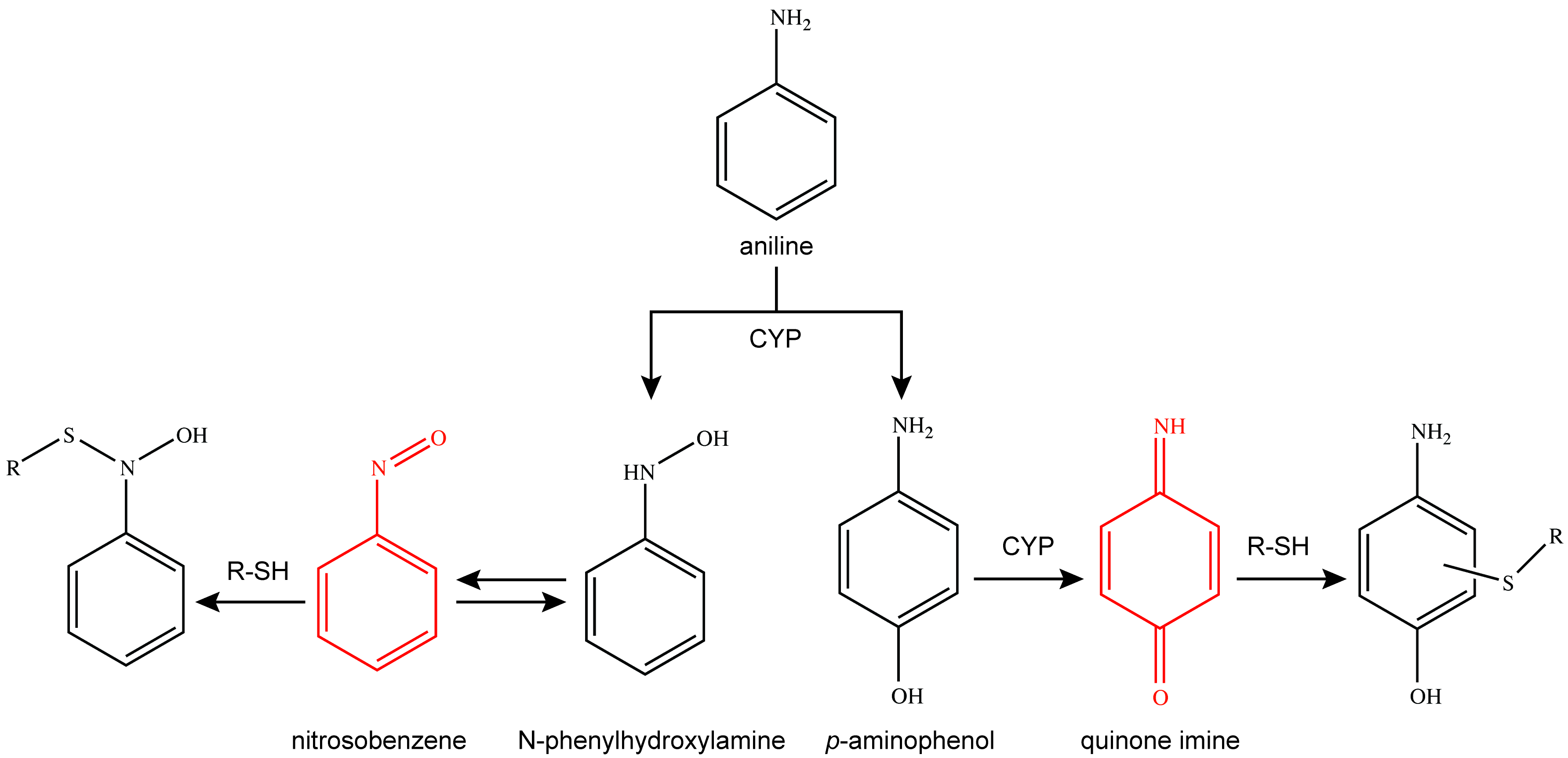

Supplement: Supplementary file 1 [file ijerph-18-07139-s001.zip › Supplemental Figure S4.tif]

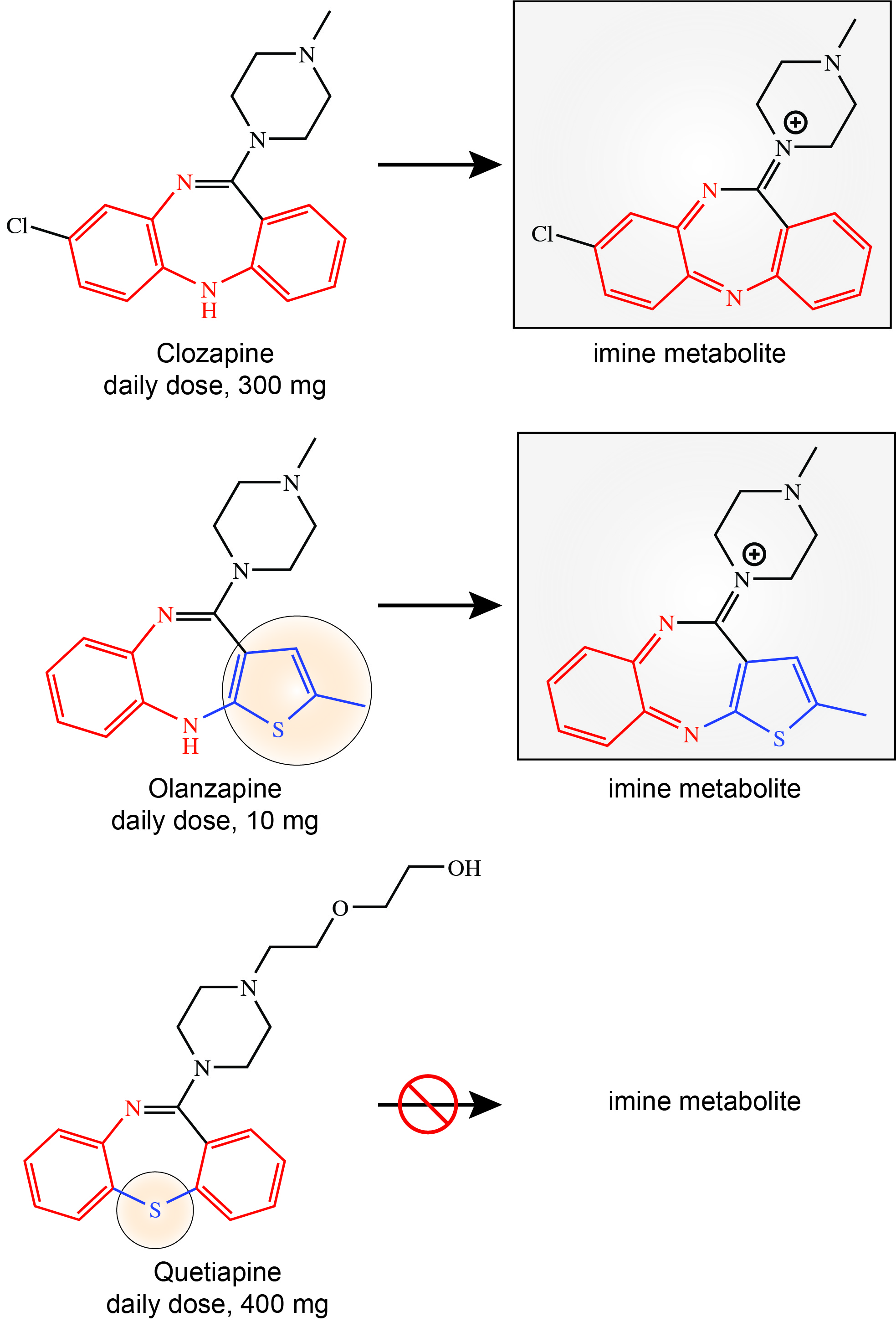

Supplement: Supplementary file 1 [file ijerph-18-07139-s001.zip › Supplemental Figure S5.jpg]

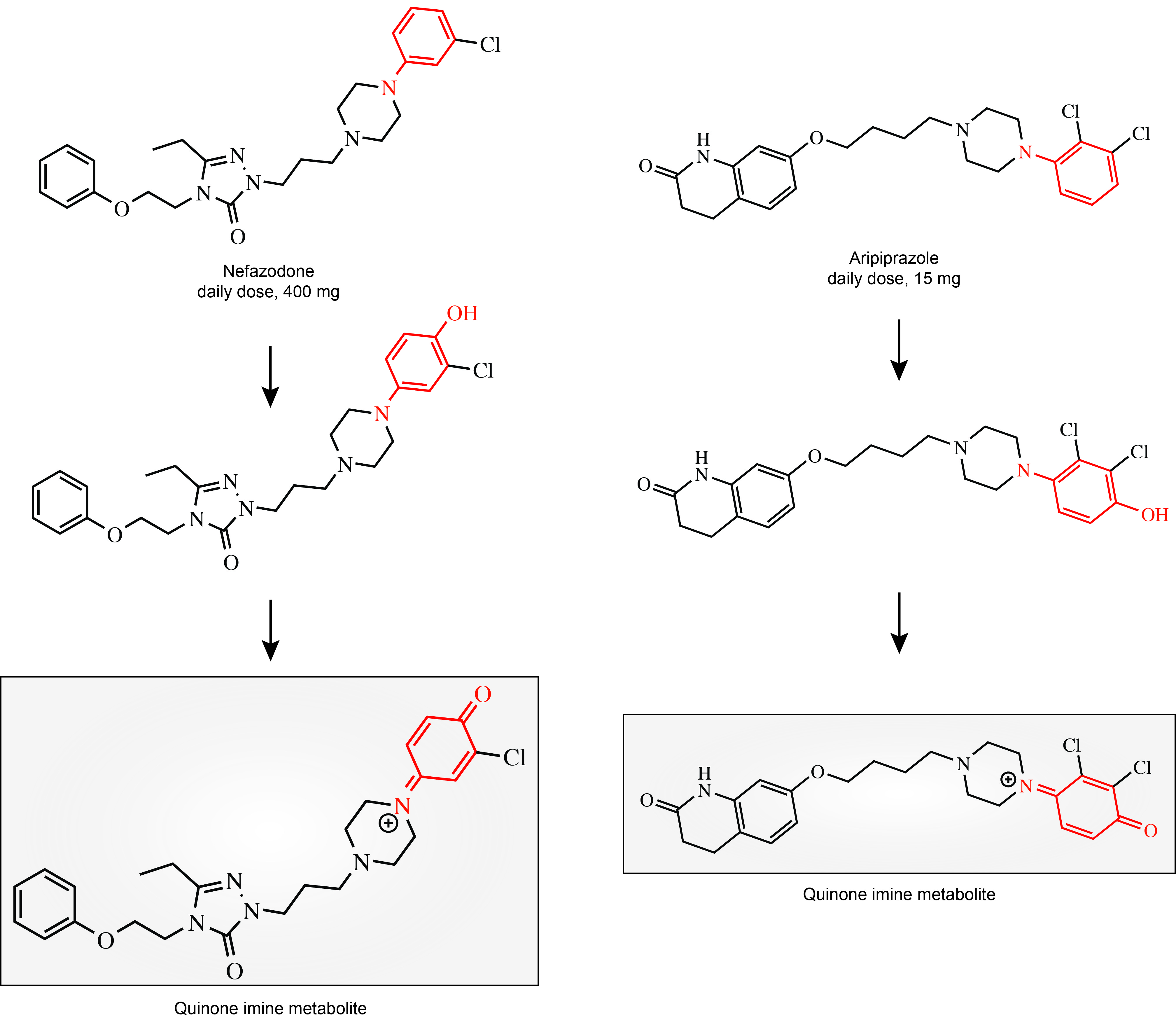

Supplement: Supplementary file 1 [file ijerph-18-07139-s001.zip › Supplemental Figure S6.jpg]
